# Accelerating Hepatitis C virus elimination in Egypt by 2030: A national survey of communication for behavioral development as a modelling study

**DOI:** 10.1371/journal.pone.0242257

**Published:** 2021-02-23

**Authors:** Ammal M. Metwally, Dalia M. Elmosalami, Hazem Elhariri, Lobna A. El Etreby, Ahmed Aboulghate, Marwa M. El-Sonbaty, Amira Mohsen, Rehan M. Saleh, Ghada A. Abdel-Latif, Sahar Samy, Sherif E. El Deeb, Asmaa M. Fathy, Mohab M. Salah, Mohamed A. Abdel Mawla, Hanaa M. Imam, Nihad A. Ibrahim, Fatma A. Shaaban, Reham Y. Elamir, Mohamed Abdelrahman, Manal H. El-Sayed

**Affiliations:** 1 Community Medicine Research Department, National Research Centre, Dokki, Cairo, Egypt; 2 Medical Research Division, Child Health Department, National Research Centre, Dokki, Cairo, Egypt; 3 Department of Pediatrics, College of Medicine, Taibah University, Madinah, KSA; 4 Epidemiology Dept., Ministry of Health and Population, Cairo, Egypt; 5 Pediatrics Dept., National Research Centre, Dokki, Cairo, Egypt; 6 Skin and Venereal Diseases Research Dept., National Research Centre, Dokki, Cairo, Egypt; 7 Community Medicine Dept., Faculty of Medicine, Cairo University of Egypt, Giza, Egypt; 8 Department of Pediatrics and Clinical Research Center, Faculty of Medicine, Ain Shams University, Cairo, Egypt; Centers for Disease Control and Prevention, UNITED STATES

## Abstract

**Aim of the work:**

This study aimed at assessing the dominance of risk practices associated with HCV endemicity in Egypt and detecting the behavioral development level concerning different aspects of HCV risk behaviors with respect to age and gender. The survey highlights the most cost-effective strategies that could accelerate HCV elimination in Egypt.

**Subjects and methods:**

A national household survey targeted 3780 individuals (age range: 10–85 years). The sample was a systematic probability proportionate to size from 6 governorates representing the six major subdivisions of Egypt. The indicators used for assessing the behavioral development level towards HCV included six domains: awareness (7 indicators), perceived risk (5 indicators), motivation with the intention to change (4 and 5 indicators for males and females respectively), trial, rejection or adoption (6 and 5 indicators for males and females respectively).

**Results:**

The study revealed that along the continuum of behavior development, the percentage of the participants who acquired half of the scores was as follows: 73.1% aware, 69.8% developed perceived risk, 80.6% motivated with only 28.9% adopting the recommended behaviors, 32% rejected them, 2.3% were in the trial stage versus 35.8% who did not try any. Adolescents had significantly lower levels of development for almost all domains when compared to adults. Statistical higher significance was detected in favor of adults, employees, married, Lower Egypt governorates, and university-educated participants (p<0.001) regarding awareness, perceived risk, and motivation scores. More than half of the participants incorrectly believed that contaminated food, sharing food utilities, contaminated water, mosquitoes, and schistosomiasis would lead to HCV transmission.

**Conclusion:**

Egypt would be closer to HCV elimination when cost-effective strategies are directed not towards creating awareness, perceived risk or motivation to change- (at an acceptable level)- but towards motivating adopting risk-reduction behaviors for HCV, tackling misconceptions and reinforcement of social support.

## Introduction

The Egyptian government has taken successful measures to control Hepatitis C Virus (HCV) as a public health threat, with a high potential for eliminating this disease before 2030. Efforts for achieving this goal should not be limited to testing and treatment only, but should also target the level of behavioral development that is still defective.

The World Health Organization (WHO) has recently formulated the ‘Global Health Sector Strategy on Viral Hepatitis, 2016–2021 with service coverage targeting to eliminate HCV as a public health threat by 2030 [1, 2]. Action to combat viral hepatitis has now been integrated into the United Nations’ 2030 Agenda for Sustainable Development [3].

One of the countries that was most affected by hepatitis C virus (HCV) is Egypt. According to the data of the Egyptian Health Issues Survey (EIHS), 14.7% of the people aged 15–59 years had an active hepatitis infection in 2009 [4], which decreased to 7% in 2015 [5] and was substantially higher than global levels [6]. To face this challenge, Egypt developed a national strategy for HCV control and established HCV prevention and treatment programs. This strategy covers six main components of prevention and control: surveillance, infection control, improving blood safety, hepatitis B vaccination, health education to providers and communities, and care and treatment [7, 8].

Scientific research clearly shows that personal health behaviors played a major role in endemicity of hepatitis, mainly hepatitis C in Egypt [9–12].

Prevention is the only safeguard against epidemics of viral hepatitis. Knowing facts, having proper attitudes and adopting certain behaviors are critical in preventing the spread of HCV in Egypt [13]. However, little is known about the extent to which Egyptians are participating in practices that limit the spread of HCV and their level of awareness. There have been very few attempts to evaluate the Knowledge, Attitudes and Practice (KAP) regarding HCV infection [14].

Communication for development (C4D) approach was designed to help meet national development goals aiming at promoting and solving health problems. C4D goes beyond collecting information about people to understand their beliefs, social and cultural norms to communicate with them at best. C4D is a two-way process for using the gained information to set strategies, take actions and implement programs that empower individuals and communities to improve their lives [15, 16].

The current study was a national formative research survey aiming at assessing the dominance of risk practices associated with HCV endemicity in Egypt to decide which practices could be targeted and identify the behavioral development level concerning different aspects of HCV risk behaviors with respect to age and gender. The survey highlights the most cost-effective strategies that could accelerate HCV elimination in Egypt.

## Methods

### Study design and settings

A cross sectional national formative research survey was conducted during a period of two years starting from November 2014 till November 2016. The national survey was directed to randomly selected families from six governorates representing the six major subdivisions of Egypt; Urban governorates (Cairo), rural and urban Upper Egypt (Beni suef and Assuit), urban and rural Lower Egypt (El Dakahlya and El Gharbya) and the Frontier governorates (Red Sea).

### Ethical approval

The study was approved by the Ministry of Health and Population (MOHP) and the Medical Research Ethics Committee of the National Research Centre with ethical approval number of 10120. A written informed consent was taken from every interviewee. Consent from parents or guardians of the minors included in the study was also obtained. The conduct of the study complied with the International Ethical Guidelines for Biomedical Research Involving Human Subjects [17].

### Study sample frame

Three sampling frames were chosen;

**The first sampling frame** used was the comprehensive list of the governorates in Egypt according to the enumeration census from Central Agency for Public, Mobilization and Statistics (CAPMAS) 2014. The choice of governorates was representative of the Six Urban Governorates and the 18 governorates in Lower Egypt, Upper Egypt and the five frontier/ desert governorates.

**The second frame** used was the choice of the districts by the use of a self-weighting implicit stratification sample; it is a form of geographic stratification that is used together with systematic probability proportionate to size (PPS). The number of individuals selected per district was proportional to their population in which sampling automatically distributes the sample proportionately into each of the nation’s administrative subdivisions, as well as the urban and rural sectors. Accordingly, five districts were selected from Cairo governorate, three from El Dakahlya governorate, three from El Gharbyia governorate, three from Assuit governorate, three from Beni suief governorate and one from Red Sea governorate.

**The third stage** followed the same methodology of the second stage for each district. The **use of 50-PSU** cluster ensured both the adequate sample size and heterogenecity of data collected. The following numbers of clusters were randomly selected within the selected district; Cairo (12), El Dakahlya (12), El Garbya (8), Assuit (8), Beni suief (6) and Red Sea (4).

### Survey sample size

A sample size of 3430 produces a two-sided 98% confidence interval with a width equal to 0.040 when the sample proportion is 0.500 [18]. With the addition of 10% expected losses, the required sample size equals 3773 to be rounded to 3780. The sample was distributed as follows: 35.5% from Upper Egypt, 35.3% from Lower Egypt, 21.3% from Cairo and 7.5% from Frontier governorates. Participants were classified into two groups according to their distribution in the community; adolescents between 10–19 years (n = 960) and adults above 19 years (n = 2820). According to the WHO the adolescent is defined any person between ages 10 and 19 years [19].

### Study instruments

#### Formative research

The level of recommended behavior development towards eradication of HCV, dominance of current risky behaviors and wrong beliefs regarding HCV were assessed by formative research. This was done by Ministry of Health personnel supervised by a professional team from the National Research Centre.

A questionnaire was constructed to identify the level of behavioral development. The questionnaire was designed to cover awareness, attitude and risk behavior related questions. The questions included symptoms, complications, methods of HCV transmission, prevention, and treatment of HCV. The level of behavioral development/change was determined. A written informed consent was obtained from adult interviewees and the parents/guardians of minors included in the study. The questionnaire is included in the Supporting files of this paper both in English (S1 Questionnaire) and the original language (Arabic) (S2 Questionnaire).

The questionnaire was reviewed and revised after being piloted on 50 participants to assure its content, validity, accuracy and clarity of items. Based on the results of the pilot study, the questionnaire was modified and made ready for use. The final version of the questionnaire contained three sections: i) the patient’s socio-demographic data including age, gender, educational level, occupation, and address to determine our sample geographical distribution; ii) the personal data and iii) the patient’s level of behavior development.

Several key performance indicators (KPIs) were used to measure the level of behavior development [20]. There were six levels of behavioral development/change; each level was explored for interviewed participants by certain indicators:

### Levels of behavior development and number of indicators per each level

1. (Awareness); Percentage who are aware of the problem; seven indicators
2. (Perceived risk); Percentage who are concerned about the problem measured by feeling at risk so acquired and internalized the knowledge; five indicators.
3. (Motivated); Percentage who are motivated and had positive attitude with intention to do something about preventing and counteracting the problem; five indicators for females and four indicators for males.
4. (Tried out recommended behaviors); Percentage who tried out the recommended safe behaviors; six indicators for males and five for females.
5. (Rejected); Percentage who rejected the recommended safe behaviors; six indicators for males and five for females.
6. (Adopted); Percentage who adopted the recommended safe behaviors; six indicators for males and five for females.

The seven awareness indicators are concerned with what is considered as potential modes of transmission or methods of prevention/ or certain activities:

1. Using previously used syringes
2. Use of non-sterilized equipment during dental procedures
3. Through needle stick injury (using one’s own needles for tattoo/hijama/acupuncture) the
4. Sharing shaving equipment or nail cutters
5. Sharing toothbrushes with other family members
6. It is better for HCV patients to get vaccinated against Hepatitis B
7. Using new syringes/or needles which have not been used before reduces the risk of becoming infected with HCV

The five perceived risk indicators:

1. HCV infection is a serious disease
2. Hepatocellular Carcinoma is a complication of HCV
3. Cirrhosis is a complication of HCV
4. Early diagnosis could make a change in patients’ health
5. Fatigue from the slightest effort is a symptom of HCV

The motivation indicators:

1. Not sharing nail cutters and/or scissors between family members
2. Not sharing sponges for personal cleaning
3. Not sharing the use of scarves’ pins by veiled females in the same family (only for females)
4. Not sharing shaving tools
5. Not sharing tooth brushes

Trial/rejection or Adoption of recommended safe practices:

1. Not sharing nail cutters and scissors between family members
2. Not sharing sponges for personal cleaning
3. Not sharing the use of scarves’ pins by veiled females in the same family (only for females)
4. Not sharing shaving tools
5. Not sharing tooth brushes
6. Letting the barber show them changing of the shaving tools (only for males)
7. Use of one’s own shaving instruments at the barber (only for males)

Health Education and counseling were provided to the participants after fulfillment of the questionnaires. Reduction of risky behaviors that are mostly widespread among the public were emphasized. Moreover, medical convoys were organized to the villages to manage the patients’ health status and complications through offering free medical examinations in the fields of internal medicine and pediatrics with the provision of free medication.

### Statistical analysis

The corrected answers were calculated according to each level of behavior development to provide the percentage of participants who were aware of chronic HCV infection, were concerned about HCV and acquired knowledge, were motivated and had positive attitude to follow recommended practices to eliminate HCV from the village, developed an intention to act in order to address HCV elimination, attempted and/or tried out the recommended safe behaviors, and rejected or adopted the recommended behaviors.

Scoring system was done for each of the six levels of behavior development. The right answer (or behavior) was given a score 1, the wrong answer (behavior) was given a score 0. The number of positive indicators in each domains was divided by the total indicators (with consideration to males and females specific questions), then the percentage was calculated and categorized accordingly.

The studied participants were classified into four categories according to their acquired score in each level of behavior development: <25, 25%- <50, 50%- <75%, > 75% (e.g for the awareness <25; means being aware by less than 25% of the awareness indicators, 25%- <50 means being aware by more than 25% and less than half of the awareness indicators and so on).

When 50% of the participants or more were in category one or two, it means that they are bad for that level. Whereas being in category three, it means neutral and if in category four, it means good.

Determination of strategies to be followed for the elimination of HCV is dependent on the calculation of scores of each level of the behavior development.

Statistical analysis of data was done using SPSS program (Statistical Package for Social Sciences) version 16. The responses of the interviewed participants were analyzed and demonstrated as comparisons according to their age groups and gender. Descriptive statistics (means, standard deviation, median, counts, and percentages) were used to describe the quantitative and categorical study variables. Chi square was done for data that is presented by numbers and percent. Continuous data was expressed as mean and standard deviation. Independent student t-test was used to compare between two means, and ANOVA to compare more than two means. Bonferroni Post Hoc test was used to determine from where differences truly came from. Raw data is available in S1 and S2 Raw datas

## Results

Our study population consisted of 43.9% males and 56.1% females with a mean age of 33.9±16.5 years and an age range between 10 and 85 years. One quarter (25.4%) of the participants were adolescents while three quarters (74.6%) were adults. About two thirds of the studied population (62.2%) were married. Regarding education, the highest percentage of the studied individuals were illiterate, could read and write (33.3%) which is nearly equivalent to those with secondary or equivalent education (29.5%). Those who had university or higher education were the least percentage (13.3%). About two thirds of the studied population were unemployed (Table 1).

**Table 1:** Socio-demographic characteristics of the surveyed participants (n = 3780).

Characteristics	Total (n = 3780)	
	Number	%
**Governorate:**		
**Cairo**	820	21.7%
**El Dakahlya**	790	20.9%
**El Gharbyia**	543	14.4%
**Assiut**	688	18.2%
**Beni suief**	654	17.3%
**Red sea**	285	7.5%
**Gender:**		
**Male**	1658	43.9%
**Female**	2122	56.1%
**Adolescents (10-≤19)**	960	25.4%
**Adults (>19)**	2820	74.6%
**Age:**		
**10- <20**	960	25.4%
**20- <30**	756	20.0%
**30- <40**	685	18.1%
**40- <50**	605	16.0%
**50- <60**	456	12.1%
**≥60**	318	8.4%
**Mean age**	33.94±16.519	
**Marital status:**		
**1. Single**	1256	33.3%
**2. Married**	2351	62.2%
**3. Divorced**	23	0.6%
**4. Widow**	150	3.9%
**Education:**		
**1. Illiterate**	858	22.6%
**2. Read and write**	407	10.7%
**3. Primary**	399	10.5%
**4. Preparatory**	497	13.1%
**5. Secondary or equivalent (technical-vocational)**	1116	29.5%
**6. University or more**	503	13.3%
**Employment:**		
**1. Working**	1175	31.1%
**2. Not working**	2605	68.9%

Regarding misconceptions about the methods of HCV transmission, more than 50% of the participants incorrectly believed that contaminated food (59.3%) and water (65.7%), sharing food utilities (59.1%), schistosomiasis (54.7%) and mosquitoes (50.7%) might lead to HCV transmission. Females had significantly more misconceptions as compared to males (p<0.001) (Table 2).

**Table 2:** Misconceptions about practices leading to HCV transmission.

Misconceived practices	Adolescents	Adults	Males	Females	Total
	N = 960	N = 2820	N = 1658	N = 2122	N = 3780
	N((%	N(%)	N(%)	N(%)	N(%)
Contaminated food	639(66.6)	1859(65.9)	983(59.3)	1515(71.4)**	2498(66.1)
Sharing food utilities	604(62.9)	1631(57.8)*	915(55.2)	1320(62.2)**	2235(59.1)
Cough and sneezing	465(48.4)	1075(38.1)**	591(35.6)	949(44.7)**	1540(40.7)
Hand shaking and hugs	299(31.1)	566(20.1)**	331(20.0)	534(25.2)**	865(22.8)
Mosquitos	395(41.1)	1522(54.0)**	772(46.6)	1145(54.0)**	1917(50.7)
Schistosomiasis	459(47.8)	1610(57.1)**	881(53.1)	1188(56.0)	2069(54.7)
Contaminated water	623(64.9)	1863(66.1)	1018(61.4)	1468(69.2)**	2486(65.7)
Air born infection in public places	482(50.2)	1181(41.9)**	645(38.9)	1018(48.0)**	1663(43.9)
Fatty meals	234(24.4)	752(26.7)	349(21.0)	637(30.0)**	986(26.0)
Sexual relations	170(17.7)	1023(36.3)**	508(30.6)	685(32.3)	1193(31.5)
From HCV infected pregnant mother to her fetus	364(37.9)	1354(48.0)**	712(42.9)	1006(47.4)*	1718(45.4)
From HCV infected mother to her infants during breast feeding	284(29.6)	1081(38.3)**	568(34.3)	797(37.6)*	1365(36.1)

*Significant,

**Highly significant.

The six domains concerning the continuum of behavioral development level are shown in Table 3. Regarding the mean scores of the six levels of behavioral development, the awareness score, the perceived risk score and the motivation score were high (60.9±27.5, 65.8±31.9 and 65.7±30.4 respectively). The mean score was <40% regarding rejection, followed by adoption, and the least was the trial (7.7±13.3). However, the mean score of the adoption was only 31.5±28.6. Meanwhile, the awareness score, the perceived risk score and the motivation score were significantly higher among adults, employed, married, Lower Egypt governorates and university educated participants (p<0.001) (Table 3).

**Table 3:** Comparison of mean scores of the six domains of behavior development level of participants based on socio-demographic characteristics.

		Awareness score	Perceived risk score	Motivation to do recommended behaviors score	Trial of recommended behaviors score	Rejection of recommended behaviors score	Adoption of recommended behaviors score
**Gender**	Males	60.2±27.6	65.2±32.4	70.8±29.4	9.3±15.3	36.9±17.5	27.0±23.4
	number of indicators	7	5	4	6	6	6
	Females	61.4±27.5	66.3±31.5	61.7±30.5**	6.5±11.3**	37.4±19.1	35.0±31.7**
	number of indicators	7	5	5	5	5	5
**Age groups**	Adolescents	53.0±28.9	55.5±33.3	59.2±32.6	8.4±13.8	37.1±18.5	27.2±28.8
	Adults	63.6±26.5**	69.4±30.6**	67.9±29.2**	7.5±13.1	37.1±18.4	32.9±28.4**
**Employment**	Working	64.7±25.5	72.1±29.8	73.0±26.8	7.7±14.0	37.1±17.6	31.0±24.9
	Not working	59.1±28.2**	63.0±32.4**	62.4±31.3**	7.7±13.0	37.1±18.7	31.7±30.1
**Marital Status**	Married	63.7±26.0	70.2±30.4	67.9±29.1	7.4±13.0	37.1±18.4	32.7±28.4
	Not	56.2±29.2**	58.7±33.0**	62.0±31.9**	8.2±13.8	37.1±18.4	29.3±28.8**
**Main 4 geographical regions**	Middle	63.7±26.4	65.1±31.1#$	68.3±29.5	5.8±11.5	35.4±17.9#	32.5±29.8
	Frontier	63.4±24.7	64.2±29.2€	68.6±26.7	4.7±9.4	37.0±19.4	31.5±30.1
	Upper	54.4±30.8#^∞	59.9±34.3∞	58.1±32.7#^∞	10.9±15.2#^∞	38.3±18.4	28.5±27.6 ∞
	Lower	65.1±23.8**	72.6±29.0**	71.0±27.5**	6.4±12.3**	37.0±18.5*	33.8±28.3**
**Education level**	-Illiterate	53.6±31.5abc	57.4±34.2abc	58.6±33.2 bc	11.1±14.9 abc	37.9±18.6	32.1±29.0
	-Read, write, primary	54.8±28.6 de	58.4±34.1 de	59.8±31.6 de	9.6±14.7 ie	35.9±18.8 i	30.5±28.6
	-Preparatory	57.8±27.8fg	62.3±31.8 fg	62.8±31.4 fg	7.3±13.6 g	39.0±17.8	26.8±27.7 fg
	-Secondary	66.6±23.4h	72.8±27.9h	71.8±27.0 h	5.5±11.1	36.8±18.0	32.9±28.2
	-University or above	73.2±18.2**	80.2±23.1**	76.3±22.7**	4.4±10.0**	36.9±18.9*	33.4±29.3**
**Total score**		60.9±27.5	65.8±31.9	65.7±30.4	7.7±13.3	37.1±18.4	31.5±28.6

*Significant,

**Highly significant,

# significant difference between middle and upper,

$ significant difference between middle and lower,

^ significant difference between frontier and upper,

€ significant difference between frontier and lower,

∞ significant difference between upper and lower.

a significant difference between illiterate and preparatory, b significant difference between illiterate and secondary, c significant difference between illiterate and university, d significant difference between read, write and secondary, e significant difference between read, write and university, f significant difference between preparatory and secondary, g significant difference between preparatory and university, h significant difference between secondary and university, i significant difference between read, write and preparatory.

Table 4 presented community level of practice to certain selected behaviors. The highest self-reported behavior that was rejected by >60% of the participants was not sharing nail cutters (73.8%) followed by not sharing sponges for personal cleaning (63.1%). Regarding adoption, <40% adopted the assessed recommended behaviors. The highest self-reported adopted safe behavior by males was using their own shaving instruments at the barber (67.1%), and by females was not sharing scarves pins (36.9%). In the trial stage, the highest self-reported behavior was asking the barber to change shaving tools by males followed by not sharing toothbrushes. The highest undetermined behaviors to seeking trial were asking barbers to change shaving tools (72.5%) followed by not sharing shaving tools (65.7%). Generally, adults and females significantly show more adoption of most of the safe behaviors when compared to adolescents and males respectively.

**Table 4:** Assessment of the level of practice according to certain selected community behaviors.

Items	Grade	Total	Sex		Age	
			Male	Female	Adolescent	Adult
**Not sharing nail cutters**	Try	74 (2.0)	30 (1.8)a#	44 (2.1)a	24 (2.5)	50 (1.8)
	Reject	2789 (73.8)	1345 (81.1)a	1444 (68.0)b**	686 (71.5)	2103 (74.6)
	Adopt	917 (24.3)	283 (17.1)a	634 (29.9)b**	250 (26.0)	667 (23.7)
	P-value		<0.001**		0.102	
**Not sharing sponges**	Try	102 (2.7)	36 (2.2)a	66 (3.1)a	35 (3.6)a	67 (2.4)b*
	Reject	2384 (63.1)	1157 (69.8)a	1227 (57.8)b**	632 (65.8)a	1752 (62.1)b*
	Adopt	1294 (34.2)	465 (28.0)a	829 (39.1)b**	293 (30.5)a	1001 (35.5)b*
	P-value		<0.001**		0.004*	
**Not sharing scarves’ pins**^1^	Try	195 (9.2)		195 (9.2)	68 (13.0)a	127 (7.9)b**
	Reject	1143 (53.9)		1143 (53.9)	324 (61.8)a	819 (51.3)b**
	Adopt	784 (36.9)		784 (36.9)	132 (25.2)a	652 (40.8)b**
	P-value				<0.001**	
**Not sharing shaving tools**^4^	Didn’t try	2186 (65.7)	920 (63.7)a	1266 (67.2)b*	317(62.4%)	1869(66.3%)
	Try	154 (4.6)	119 (8.2)a	35 (1.9)b**	28 (5.5%)	126 (4.5%)
	Reject	191 (5.7)	179 (12.4)a	12 (0.6)b**	33 (6.5%)	158 (5.6%)
	Adopt	797 (23.9)	227 (15.7)a	570 (30.3)b**	130(25.6%)	667 (23.7%)
	P-value		<0.001**		0.352	
**Not sharing toothbrush**	Didn’t try	1878 (49.7)	900 (54.3)a	978 (46.1)b**	515 (53.6)a	1363 (48.3)b*
	Try	415 (11.0)	193 (11.6)a	222 (10.5)a	105 (10.9)a	310 (11.0)a
	Reject	193 (5.1)	100 (6.0)a	93 (4.4)b*	47 (4.9)a	146 (5.2)a
	Adopt	1294 (34.2)	465 (28.0)a	829 (39.1)b**	293 (30.5)a	1001 (35.5)b*
	P-value		<0.001**		0.025*	
**Ask barber to change shaving tools in front of them** ^2^	Didn’t try	1202 (72.5)	1202 (72.5)		316 (72.5)	886 (72.5)
	Try	271 (16.3)	271 (16.3)		67 (15.4)	204 (16.7)
	Reject	111 (6.7)	111 (6.7)		35 (8.0)	76 (6.2)
	Adopt	74 (4.5)	74 (4.5)		18 (4.1)	56 (4.6)
	P-value				0.559	
**Use of their own shaving instruments at the barber** ^2^	Didn’t try	344 (20.7)	344 (20.7)		177 (40.6)a	167 (13.7)b**
	Try	64 (3.9)	64 (3.9)		36 (8.3)a	28 (2.3)b**
	Reject	137 (8.3)	137 (8.3)		57 (13.1)a	80 (6.5)b**
	Adopt	1113 (67.1)	1113 (67.1)		166 (38.1)a	947 (77.5)b**
	P-value				<0.001**	
**Check use of sterilized tools at dental clinic**^3^	Try	143(6.2)	65(6.6)a	78(5.9)a	39(8.3)a	104(5.6)b*
	Reject	940(40.5)	372(37.5)a	568(42.7)b*	219(46.4)a	721(39.0)b**
	Adopt	1238(53.3)	555(55.9)a	683(51.4)b*	214(45.3)a	1024(55.4)b**
	P-value		0.039*		<0.001**	

# Homogenous groups have same symbols based on post hoc Bonferroni test,

*Significant,

**Highly significant.

^1^ Among female respondents only,

^2^ Only among those who had visited barber (adolescent males = 436, adult males = 1222),

^3^ Only among those who had visited dentist (total = 2321, adolescents = 472, adult = 1849, Male = 992, females = 1329),

^4^ Only among those aged 15 years or more (total = 3328, males = 1445, females = 1883, adolescents = 508, adults = 2820).

**Fig 1:**
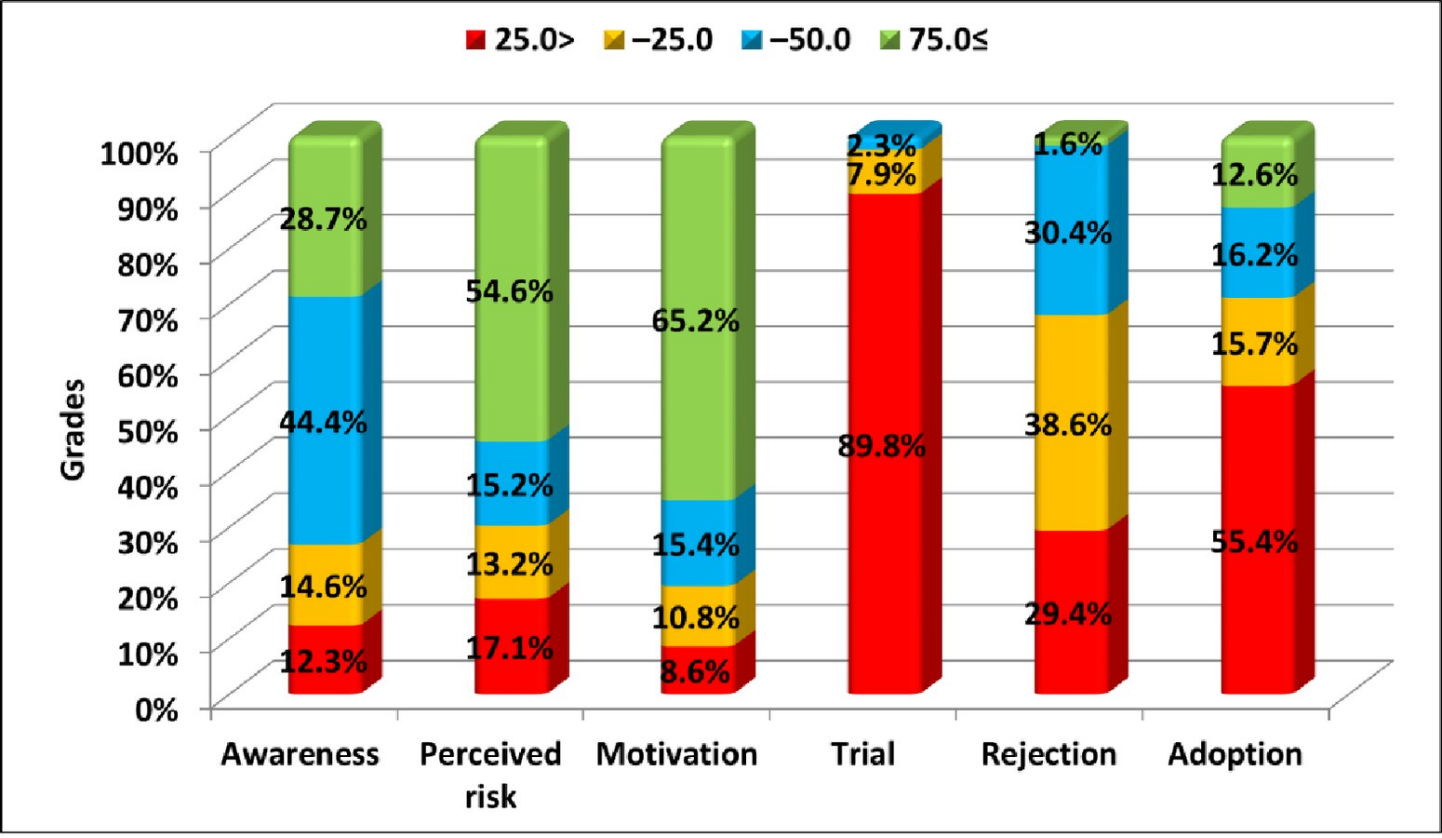
Grading scores per level of behavior development.

**Fig 2:**
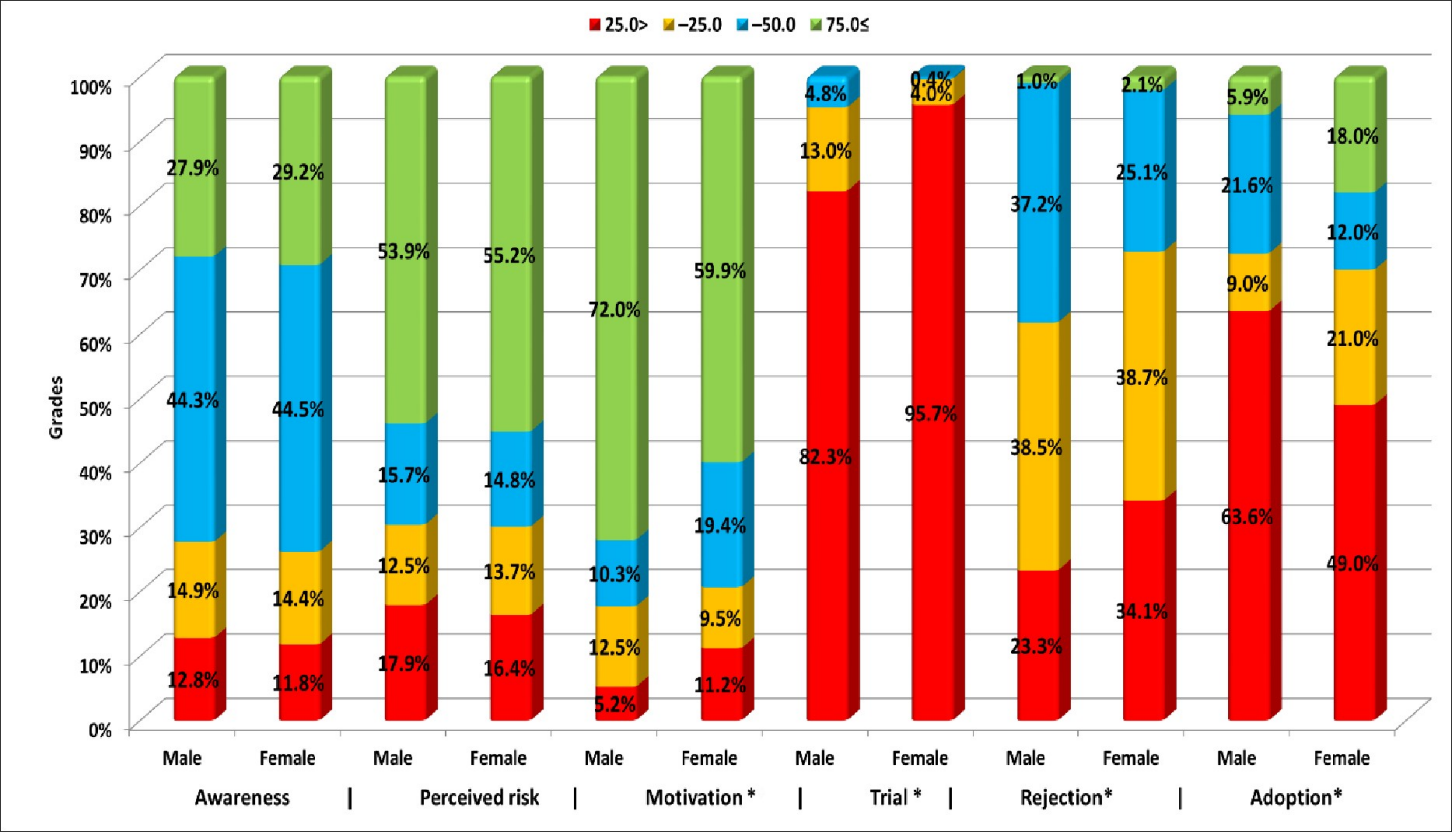
Gender differences in grading scores of behavior development levels.

**Fig 3:**
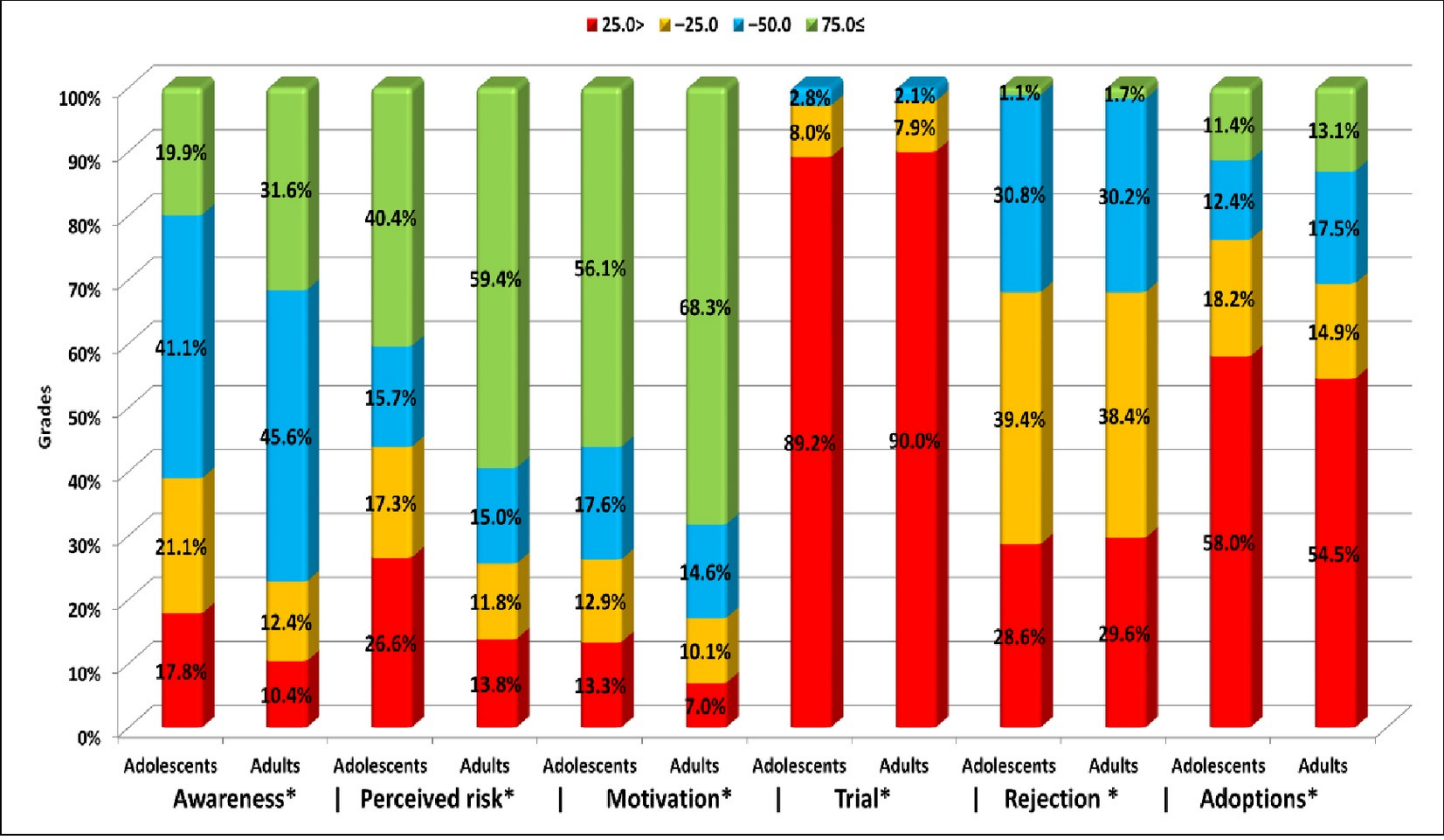
Differences between adults and adolescents in grading scores of levels of behavior development.

Around two thirds (65.2%) of the participants were motivated with the intention to follow up to 4 or 5 out of the 6 recommended behaviors for limiting the HCV problem. Meanwhile, the adoption was observed among only 12.6% of the participants versus 32% who rejected almost half of the recommended safe behaviors (Fig 1). There were no significant differences between genders in most of the levels of behavior development (Fig 2). Adults’ scores of awareness, perceived risk of HCV, motivation towards practicing safe behaviors and adoption were significantly higher than that of adolescents (p<0.001). More than two thirds (68.3%) of the adults were significantly motivated to practice more than 75% of the recommended behaviors versus 59.4% of the adolescents (Fig 3).

Table 5 provided suggested strategies to accelerate HCV elimination according to the level of behavioral development stage. Giving a high level of awareness, perceived risk, and motivation to change of participants in the current survey, strategies enhancing trial and adoption of the recommended safe behaviors are needed. Strategies towards the elimination of HCV should be directed to addressing traditional beliefs and false rumors, reinforcing safe behaviors through confidence building, and solving problems that hinder its adoption. HCV public health messages should be effectively disseminated to the target population. Media should disseminate messages that motivate adopting risk-reduction behaviors for hepatitis with special emphasis on poor and neutral behavior (< 75%) to improve the level of this behavior to be good (> 75%). Encouraging and mobilizing the community to try the recommended behaviors is required in order to engage approximately 40% of the participants in trying the recommended behaviors. This could be accelerated by identifying misbeliefs and practices to reinforce ideal ones and to adjust negative ones. Strategy of confidence building so that individuals feel and perceive that not only the community supports the recommended behavior but also the social norms and culture. At this stage multimedia channels are required e.g communities can use supportive statements and messages of respected leaders from each beneficiary and/or use of satisfied acceptors and/or individual counseling.

**Table 5:** Suggested strategies to accelerate HCV elimination according to the level of behavioral development stage.

Level of behavioral development	≥50.0%	mean score	suggested strategies (if Cutoff point median score >60)
Awareness score	2763 (73.1%)	**60.9±27.5**	• To be sustained through mass media
Perceived risk score	**2637 (69.8%)**	**65.8±31.9**	
Attitude toward not practicing risky behavior score	**3046 (80.6%)**	**65.7±30.4**	
Trial of recommended behaviors score	87 (2.3%)	**7.7±13.3**	• Encouraging and mobilizing the community to try the recommended behaviors.
			• Identifying misbeliefs and practices to reinforce ideal ones and to adjust negative ones.
			• Strategy of confidence building
Rejection of recommended behaviors score	1209 (32.0%)	**37.1±18.4**	• Strategy of problem solving and creating opportunities to continue behavior by availability of resources to monitor and reinforce recommended behaviors, skills and practices
Adoption of recommended behaviors score	1091 (28.9%)	**31.5±28.6**	
			• Strategy of reinforcement of social supports
			• Practice recommended behavior becomes self-rewarding OR external rewards are suggested temporarily
• All through behavioral development stages, services like testing, treatment care have to be affordable, available and accessible. Also, the people offering services have to be competent, approachable and welcoming			

Strategy of problem solving and creating opportunities to continue and sustain the recommended behaviors to decrease the chance of rejection have to be implemented. Availability of resources to monitor and reinforce recommended behaviors, skills and practices have to be ensured. Individual counseling and psychological rewarding of the recommended behaviors have to be used to sustain the change to the safe practices. Local community members serve as support groups, providing both information about the virus and emotional support.

## Discussion

Egypt has had the highest prevalence of HCV infection worldwide for years but has taken the largest step towards the elimination of the disease so far, with the highest treatment campaigns in the world using highly effective regimens of the direct-acting antiviral therapy resulting in mass treatment of nearly two and a half millions of Egyptian HCV patients [21].

More than 2 million individuals have been treated between 2014 and 2017. However, finding individuals unaware of their HCV infection remained a challenge. This pool of undiagnosed individuals, if untreated, are at risk of progressive disease and a potential source for infection [22]. This led eventually to a country state-sponsored mass screening campaign initiative that targeted the whole adult population. Between October 2018 and April 2019 almost 50 million of the population were tested and those positive for HCV were referred to treatment [23]. However, an essential step in elimination of HCV and its sustainability is prevention of transmission largely related to vigilant infection control practices which necessitates awareness, attitude, and adoption of safe practices.

In recent years, there has been increased an interest in application of behavior change theories in the areas of health, education, criminology, energy and international development with the hope that understanding behavioral change will improve services offered in these areas. These theories generally aim at understanding and changing a given behavior [24]. The most prevalent are learning theories), social cognitive theory, theories of reasoned action and planned behavior, trans-theoretical model of behavior change, the health action process approach and the health belief model of behavior change [25].

Success in achieving global developmental goals was dependent largely on the extent to which national planning processes are informed by all sectors of its citizens [26]. The current study applied the process of C4D which starts with informative research for determining the level of behavioral development; including rich and underprivileged, marginalized people, and those with different levels of education. Therefore, C4D could be seen as a supporting tool that can be applied to accelerate and enhance the HCV elimination.

Regarding misconceptions about exposures and practices that could lead to HCV transmission, the participants had some incorrect beliefs about HCV infection and disease, probably from former misperceived messages. More than 60% of the participants believed that virus C is a food-borne and water-borne virus, whereas more than 40% believed that the virus could be transmitted by mosquitoes. These percentages were slightly higher than a similar previous study in Egypt where participants were surveyed about their health beliefs regarding HCV [9] but much higher than a study from Pakistan where less than 15% of the participants stated that water, food, and mosquitoes were methods of transmission [27]. This may be explained by that one third of the population (33.3%) were either illiterate or just read and write. Other misconceptions that could transmit HCV in the current study included sharing food utilities among 55.2% of females and 62.2% of males. In another study, sharing food utilities was wrongly believed as a route of HCV transmission by more than 80% of participants but was reduced to less than 10% after educational campaigns to raise awareness were conducted [14]. More than one-third of the current participants wrongly believed that coughing and sneezing could transmit HCV. The Public health association in Canada stated that Hepatitis C cannot be spread by natural contact, such as kissing, hugging, or by being with someone who is coughing or sneezing. The virus is not present in food or water [28]. Combating misconceptions about HCV is important. Otherwise, dissemination of wrong information about HCV may lead to unnecessary isolation, which may lead to social stigma and depression in infected patients.

One-third (33.3%) of the participants in the current survey were either illiterate or could read and write where more than 50% of the illiterates were from Egypt upper governorates, and only 13.3% had university level of education where more than two thirds (69.6%) were from Middle and Lower governorates. However, it was found that the awareness, perceived risk and the motivation mean scores were more than 60%. Good awareness and high motivation of the current participants is most probably related to the previous community based educational programs [14] and TV mass media awareness campaign in Egypt over several years, complementing the national program for control of viral hepatitis established in 2006 [29]. On the other hand, studies done in China [30], Korea [31], Turkey [32], and Spain [33] showed poor knowledge in the community regarding HCV transmission.

Although, the awareness score, the perceived risk score and the attitude score were high (60.9±27.5, 65.8±31.9 and 65.7±30.4 respectively), the mean score of the adoption was only 31.5±28.6. This is most probably because most of the studied population were socially underprivileged. Hence, they could not afford many of the personal hygienic products. The reality of low income is that it restricts people’s options, leaving them caught between eat or be clean. So low adoption of the safe recommended behaviors in the presence of high awareness and positive attitude is most probably because of not being able to buy a private toothbrush, toothpaste or shaving tools. Therefore, they prefer to share these tools to save money.

Adults’ scores of awareness, perceived risk of HCV, motivation and adoption were significantly higher than that of adolescents (p<0.001). This is most probably due to past educational campaigns [28]. These findings suggest that adolescents may not be aware of the risk of HCV infection and highlight the urgent need for HCV behavioral interventions to reduce ongoing high-risk behavior that perpetuates the risk of HCV transmission. At the same time, for most of the behavioral development levels, males and females were similar. This could be explained by being direct relatives or close neighbors or married. Besides being living in the same environment, they are of a similar socioeconomic standard, hold close levels of education and culture, and are exposed to the same external influences.

The results of the current study were different from another study done in Egypt by Metwally et al., [9] where the percentage of those who tried and adopted the recommended behaviors was statistically higher than those who rejected these behaviors. Also, the percentage of those who rejected the safe behavior of not sharing nail cutters (73.8%) was much higher than another study done by Metwally et al., [10] where only 26.5% of HCV-ve contacts and 29.3% of HCV+ve cases reported sharing nail cutters. The differences are because the old study was conducted on HCV patients who took precise information from doctors and nurses about the importance of following and adopting the recommended behaviors that counteract HCV transmission.

Shaving at the barbershops is a well-known risk factor for HCV infection [34]. Barbers in resource-limited countries are usually unaware of the concept of blood-borne transmission, and razors and scissors are used repeatedly for different customers without intervening sterilization [35]. In the current study, only 4.5% of the males adopted the safe behavior of asking the barber to change shaving tools in front of them. This might be explained by the fact that 67.1% of the males used their own shaving instruments at the barber which is good. Meanwhile, legislations are required to enforce the sterilization of barbers’ equipment.

Toothbrushes are essential to maintain oral hygiene and health [36]. However, sharing toothbrushes may carry a risk of infection [37, 38]. The presence of injuries in the oral cavity associated with the absence of biosafety practices and the presence of contaminated blood during dental treatment could also be a risk factor for HCV transmission [39]. However, the present study showed an alarming situation where nearly half of the participants did not determine whether they would or wouldn’t share toothbrushes and only 53.3% checked the use of sterilized tools at dental clinics in spite of their high awareness and motivation score.

Regarding certain community practices, the results of this survey showed that the highest percentage for those who did not try the recommended behaviors was observed regarding asking the barber to change the shaving tools in front of them (72.5%) and not sharing shaving tools (65.7%). Surprisingly, the study done by Shiha and his colleagues [14] reported that the percentage of participants who were using their own shaving instruments at the barber shop and not sharing shaving tools increased to 75% or more as a result of behavioral intervention, indicating the powerful role of communication for behavioral change. Therefore, the behavior of not asking the barber to change shaving tools in front of them in the current study can be substituted by the behavior of using the persons own shaving instruments at the barber shop as a modified good behavior. The highest percentage that is still at the trial stage was observed for asking the barber to change shaving tools in front of them (16.3%) indicating that this behavior needs supportive strategies for engaging people to try and support its adoption.

A very low level of behavioral adoption indicators (< 25%) was observed regarding not sharing personal tools (nail cutters and shaving tools (24.3% and 23.9% respectively). The highest rejection was observed for some of the family behaviors like not sharing nail cutters (73.8%), not sharing sponges (63.1%), not sharing scarves’ pins for females (53.9%) and for checking the use of sterilized tools at dental clinics (40.5%). In the current study, we focused on behaviors like sharing scarves pins and sponges for personal cleaning among the community. These behaviors were uncovered during the group discussions in a recent study [10] and considered new emerging behaviors carrying a potential risk for HCV infection. They are also culturally sensitive to the Egyptian people and might be behind HCV endemicity. Sharing scarves’ pins by veiled women, which might be contaminated with the blood of HCV infected women, might expose the non-infected women to HCV infection on piercing the scalp or skin. Also sharing the sponge for personal cleaning might expose the non-HCV infected person to infection if the used sponge was contaminated with HCV-infected blood and penetrated any injured skin especially in rural areas with an inadequate amount of water [10].

The current study provides a cost-effective model through which behavioral related problems within low and low-middle-income communities should be tackled. The suggestions emerging from this survey can support the national program in accelerating elimination of HCV and may provide a model for other resource-limited settings with high infection rates. The suggested strategies will result in shifting the level of behavioral development for the low indicators to be neutral i.e. from < 50% to be 50%-75% and the neutral ones to be good (more than 75%).

The suggested strategies are focus on identifying misconceptions and wrong practices, to reinforce ideal ones and to adjust negative ones. Wrong beliefs essentially addressed and corrected included that virus C infection is a food-borne virus that could be transmitted by mosquitoes, could be caused by bilharziasis and could be treated by herbs. This strategy will prevent stigmatization of infected populations, thus they will not miss the opportunities for prevention, testing and treatment which will limit the endemicity of HCV. This will not happen unless misconceptions are corrected. This could be achieved by using focus groups to tackle misconceptions and reinforce social support. Also the use of multimedia could be useful in disseminating detailed information about HCV in order to modify public behavior, dispel rumors, counter myths, and correct misinformation.

Generally speaking, tackling wrong traditional beliefs has to be a prime target in any health intervention program to have a positive impact on the population. Community-based studies provide accessibility to local resources to monitor and reinforce recommended behaviors, skills, and practices. Accordingly, this is should be a way to tackle behavioral related problems within low-income communities. Many community-based studies that were dependent on communication for behavioral development and initially determined communities’ levels of behavioral development were done in Egypt. They were very successful in creating behavior changes aiming at counteracting wrong believes not only for HCV related behaviors [14] but providing support for many other health problems. Successful models of Egyptian studies range from hygiene promotion [40–42], promoting school and breastfeeding [43, 44] promoting safe motherhood and reducing maternal mortality [45, 46], supporting child psychosocial development [47, 48] alleviating health problems like diabetes [49], HBV and vaccination [50, 51], and supporting end-stage renal disease Egyptian patients [52]. Such approach was also successful for sensitive issues like accepting organ donation and resulted in shifting the attitude of almost half of the participants who were refusing posthumous organ donation to become an acceptable issue [53]. A worthy note is that all through stages, services like testing, treatment and care have to be affordable, available and accessible as recommended by patient groups studies from 25 studied countries [54]. In addition, the people offering services have to be competent, approachable and welcoming. Focusing on the social acceptability of some recommended behaviors has to be promoted; for example, using one’s own instruments at the barber/hairdresser and emphasis on the availability sterilized equipment for dental treatment or using one’s own toothbrushes, shaving brushes, and syringes. If the problem is due to unavailability of facilities, it is advisable to provide the missing equipment and devices to improve adoption and decrease rejection level of the recommended behaviors. The recommended behavior has to be rewarded (self-rewarded or externally rewarded) temporarily to sustain safe behaviors (e.g school campaigns and provision of low cost or free personal tools e.g toothbrushes, toothpaste, shaving tools, nail cutters and scarves pins). A study by Michie and colleagues concluded that recognizing the nature of what motivates persons and the economic and social pressures that act upon them will help improve public health behaviors [55].

In conclusion, it is clear that new therapies will not control the disease unless accompanied by increasing levels of behavior development. The current study demonstrated that our participants are concerned about the HCV problem. Health messages about HCV appear to have largely been received and remembered by the adult population. There is some deficit among adolescents reflecting that they have not been effectively exposed to detailed HCV public health messages. Furthermore, misconceptions about HCV transmission is obvious among all shared participants irrespective of gender and age. However, they acquired knowledge and were motivated to try out the majority of the recommended behaviors. Awareness, perceived risk and attitude mean scores were high. On the contrary, trial and adoption of the recommended safe behaviors mean scores were low with considerable high level of rejection of almost half of the recommended behaviors. On the other hand, almost one third of the participants did not try any.

Whereas, much emphasis should be put on the poor indicators for creating messages for inducement of action for improvement, strengthening of the neutral indicators to be shifted to the good ones and maintaining the good ones should be priority actions.

With the availability of an enabling environment in Egypt, the implementation of the recommended strategies to achieve behavioral change is achievable and hence accelerate and sustain the elimination of HCV. In the light of the mass screening, diagnosis, and treatment by competent, approachable, and welcoming health personnel who are offering these services, strategies to reinforce optimizing behaviors or modify negative ones could be implemented.

### Strengths of the study

This study is characterized by being the first national study in Egypt representing eight different geographical areas with a large sample size (3780 individual) at confidence level of 97.5% and two-sided margin of error (0.02), targeting all age groups and all socioeconomic levels to explore health barriers to seek HCV elimination by 2030.

This study was the first in Egypt to apply the principles of C4D, staging behavioral development level and set cost-effective strategies for HCV elimination resulting in bringing the country closer to HCV elimination. The Egyptian government may adopt these cost and time-saving strategies at governorate and district levels through key stakeholders engagement. Such suggested approach of implementation will have more impact on the sustainability of utilization of the provided governmental test and treatment services for HCV elimination.

A key component of the current study is the placement of the Egyptian Ministry of health and population (MOHP) champions both at the policy-maker level and local community level of the respective governorates involved in the survey. The champions have become effective enabling government bodies to allow a better understanding of the community under the study and facilitated identifying the perception, needs of all sections of the society; advantaged and disadvantaged groups, educated and illiterate people. The role of the Ministry of Health and community leaders in rectifying the misconceptions laid a platform for the nationwide-wide screening campaigns in the following years.

This study is unique because most of the previous studies and researches focused only on awareness, diagnosis and treatment of HCV and not on behavioral development. However, the sustainability of elimination of HCV can only be achieved through targeting public behavior. The suggested approach if adopted by other resource-limited countries with a similar situation will accelerate the process of HCV Elimination.

### Limitation of the study

This survey was limited to investigating the level of behavioral development which has to be completed by determining the best channels of communication for applying the developed culturally sensitive educational and multimedia tools. These tools can be used to improve the adoption of the safe recommended behaviors by different sectors of the community to achieve elimination of HCV by 2030 or earlier.

## Supporting information

S1 Questionnaire Questionnaire (English). (DOCX)

S2 Questionnaire Questionnaire (Arabic). (DOCX)

S1 Raw data (XLSX)

S2 Raw data (SAV)
