# Supplementary material for: Accelerating Hepatitis C virus elimination in Egypt by 2030: A national survey of communication for behavioral development as a modelling study
Source: PLoS One. 2021 Feb 23;16(2):e0242257. doi: 10.1371/journal.pone.0242257 (PMC7901784; doi:10.1371/journal.pone.0242257)
Supplement: S1 Questionnaire — (DOCX) [file pone.0242257.s001.docx]

**Community behavioral development level questionnaire concerning different aspects of HCV risk behaviors.**


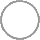

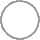

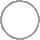

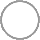

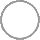

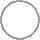

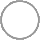

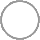

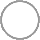


**N.B: Please refer to the consent form before fulfilling this questionnaire**

This is a personal form and will not be revealed except to the research team

| **Name of data collector Date:** |
| --- |
| **Name of administrative officer:** |
| **Start time of interview:** |
| **End time of interview:** |
| **Signature of the respondent to fill in the questionnaire** |

**Demographic data**

**Governorate: District: Village:**

**Personal data**

**Name: Age: Gender:**

**Marital status:** a-Single b-Married c-Divorced d-Widow

**Educational Status:** a- Illiterate b- Read and write c- Primary

d- Preparatory e- Secondary or equivalent f- University or more

**Occupational Status:** a-Not working (Housewife/ under age/ unemployed/ retired/ sick or disabled)

b- Working (mention….)

1. Are you a Hepatitis C patient?

Yes no

Don’t know

2. Hepatitis C is caused by:

Bacteria virus other

3. Is Hepatitis C dangerous?

Yes no

Don’t know

If yes, why?

4. In your opinion, could a Hepatitis C patient stay long without symptoms?


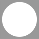
 yes
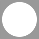
 no


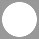
 Don’t know

5. Do you think that early detection could change the patient's health status?


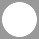
 yes
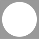
 no


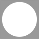
 Don’t know

6. Do you know any preventive measures?


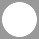
 yes
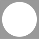
 no


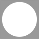
 Don't know

If yes: what are these preventive measures?

7. What are the symptoms you know?

8. What are the complications you know?

9. In your opinion, which of the following is considered a way of transmission?

Agree neutral disagree

Contaminated food


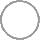

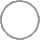

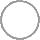

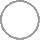

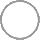

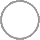

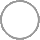

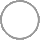

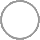

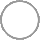

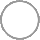

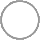

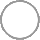

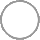

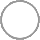


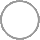

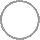

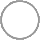
Sharing food utensils


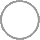

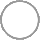

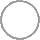


Sharing shaving equipment, nail cutters or through needle stick injury


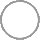

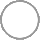

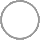
If, for example, you had an injury in your mouth, would you share your tooth brush with your family?

Sexual intercourse


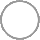

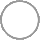

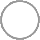
During pregnancy from mother to fetus

During lactation form mother to baby


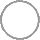

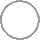

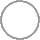

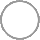

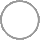

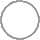
Coughing & sneezing

Handshaking & hugs Mosquito bites

Used syringes


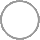

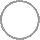

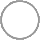
Organ transplantation Renal dialysis


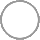

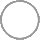

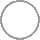


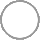

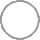

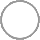
Dental care and treatment

Tattoo and Chinese needles


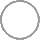

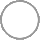

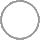

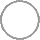

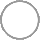

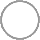


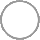

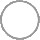
Schistosomiasis

Contaminated water

Airborne infection in

crowded places Wound infection Fatty meals Hijama


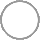

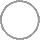

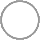

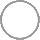

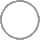

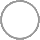

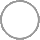

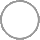

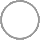


Using public toilets Using public phones

Sitting side by side in

classrooms Flies


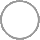

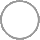

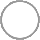


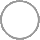

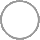

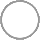

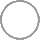

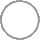

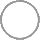

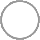

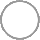

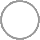

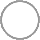

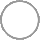

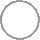

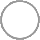

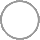

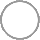
Agree neutral disagree

10. Tell me what you think about each of the following symptom of the disease:

Agree disagree neutral don't know


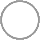

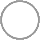

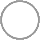

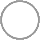
High temperature


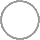
Yellowish color of eyes and skin

Right side abdominal pain

Dark urine

Joint pain

Fatigue

No symptoms

11. Tell me what you think about each of the following complication of the disease:

Agree disagree neutral don't know

Liver cirrhosis

Hepatocellular carcinoma

Esophageal varices

Abdominal Ascites

Liver failure (fatigue, weakness, malaise, laziness )

Psychological problems (depression, stress, nervousness..)

12. Tell me what you think about each of the following:

agree disagree neutral don't know

- It is better for HCV patients to be vaccinated against HAV and HBV

- Hepatitis C patients could live many years without knowing about their illness

- Using new syringes may reduce the risk of new infections with C virus

- Proper treatment could lead to complete cure and make the virus disappear from the blood

- Interferon therapy could cause depression

13. Would you do or you are actually doing each of the following?

of course no maybe no maybe yes of course yes

- Ask your barber to change the razor in front of you

- Ask your barber to use

your own shaving

machine

- Make sure that the dentist's tools are sterilized

- if you are a Hepatitis C

patient, would you

inform your dentist about your infection

-Not share tooth brush with any of the family members

-Not share scarf pins used by veiled females with any of the family members

-Not share sponge used during bathing with any of the family members

-Not share scissors or nail cutters with any of the family members

14. If a family member offered you any of the following, would you agree to use it?

of course no maybe no maybe yes of course yes

Clothes

Shaving tools

Tooth brushes

Unwashed food and drinking utensils
