# Supplementary material for: Accelerating Hepatitis C virus elimination in Egypt by 2030: A national survey of communication for behavioral development as a modelling study
Source: PLoS One. 2021 Feb 23;16(2):e0242257. doi: 10.1371/journal.pone.0242257 (PMC7901784; doi:10.1371/journal.pone.0242257)
Supplement: S2 Questionnaire — (DOCX) [file pone.0242257.s002.docx]

استبيان قياس مستوى التطور السلوكي فيما يتعلق بجوانب مختلفة من السلوكيات المحفوفة بالمخاطر لفيروس التهاب الكبد سي

**هذه الاستمارة شخصية ولن يتطلع عليها إلا الفريق البحثي**

- **اسم المحافظة:.....................................................**
- **اسم المركز: ......................................................**
- **اسم القرية: .......................................................**
- **اسم جامع البيانات: ........................................................**
- **اسم المراجع (المشرف المكتبي): .........................................**
- **وقت ابتداء المقابلة: .................................................**
- **وقت انتهاء المقابلة: ................................................**
- **التاريخ: ...............................................................**

**بيانات شخصية:**

**الأسم:................................. العمر: ......... الجنس: ...........**

**الحالة الإجتماعية:**

**ا- أعزب ب- متزوج ت- مطلق ث- أرمل**

**الحالة التعليمية:**

**ا- أمي ب- يقرأ و يكتب ت- إبتدائية**

**ث- إعدادية ج- ثانوي/ما يعادلها ح- جامعي/فما فوق**

**الحالة الوظيفية:**

**1- لا يعمل (ربة منزل- تحت سن العمل –عاطل - متقاعد – مريض أو معاق)**

**2- يعمل ( إذكر نزع العمل ...........................)**

| استمارة المعرفة |  |
| --- | --- |
| **[1]هل انت مريض بإلتهاب الكبد الوبائي سي؟1-نعم 2-لا 3- لاأعرف** |  |
| **[2]ما هو سبب التهاب الكبد الوبائي سي؟ 1-التهاب بالكبد نتيجة بكتيريا 2-التهاب بالكبد نتيجة فيروسي 3-أخرى** |  |
| **[3]ايه رأيك هل هو مرض خطير؟1- نعم 2- لا 3- لا أعرف** |  |
| **[3.1]اذا كانت الإجابة نعم: لماذا؟ لأنه يؤدي إلى: "لا تذكر الإجابة" (اكتب ما يقولة المجيب)**  **1-تليف في الكبد 2-دوالي المرئ 3-استسقاء بالبطن (ميه) 4-التهاب مزمن في الكبد**  **5-فشل في الكبد 6 -تضخم في الطحال** |  |
| **[4]ايه رأيك بعد الإصابة بالفيروس سي هل ممكن للمريض أن يبقى لفترة من غير أن يظهر عليه أي أعراض للمرض؟ 1-نعم 2-لا 3-لا أعرف** |  |
| **[5]هل تعتقد أن الاكتشاف المبكر للمرض يشكل أهمية في تغيير الحالة الصحية للمريض؟ 1-نعم 2-لا 3-لا أعرف** |  |
| **[6]هل تعرف طرق الوقاية من المرض؟1-نعم 2-لا 3-لا أعرف (في حالة الإجابة بلا او لا اعرف اذهب الى 8)** |  |
| **[6.1] اذا كانت الإجابة نعم: ما هي؟ "لا تذكر الإجابة" (اكتب ما يقولة المجيب)** |  |
| **[7] ما هى أعراض المرض الذى تعرفها؟** |  |
| **[8] ما هى مضاعافات المرض الذى تعرفها؟** |  |

| استمارة الأتجاهات | |  |
| --- | --- | --- |
| **[9] في اعتقادك أي من الطرق الآتية هي طرق العدوى للمرض؟**  **1=موافق 2= محايد (قد يحدث وقد لا يحدث) 3= غير موافق** |  | |
| **1-عن طريق الأكل الملوث مثل خضار ملوث** |  | |
| **2-عن طريق مشاركة أدوات الطعام مع المريض (الأكواب والأطباق بالمطبخ).** |  | |
| **3-عن طريق المشاركة في استخدام أدوات الحلاقة، القصافة، المقص، أو عن طريق الوغز بالإبر** |  | |
| **4- يا ترى لو عندك جرح في أي مكان في جسمك مثل جرح بالفم هل تشارك أدواتك الشخصية مع أحد من أفراد الأسرة مثل فرشاة الأسنان** |  | |
| **5-عن طريق الجماع (الإتصال الجنسي )** |  | |
| **6-من الأم للجنين أثناء الحمل** |  | |
| **7-الأطفال الرضع اثناء الرضاعة من أمهاتهم المصابين** |  | |
| **8-العطس والكحة** |  | |
| **9-عن طريق السلام باليد والمعانقة بالأحضان** |  | |
| **10-عن طريق الناموس** |  | |
| **11-عن طريق استخدام حقن تم استخدامها سابقا** |  | |
| **12-عن طريق زرع الأعضاء** |  | |
| **13-عن طريق غسيل الكلى** |  | |
| **14-عن طريق علاج الأسنان** |  | |
| **15-عن طريق الابر صينية أو الوشم** |  | |
| **16-عن طريق ديدان البلهارسيا** |  | |
| **17- عن طريق شرب مياة ملوثة** |  | |
| **18- عدوى من الهواء في الأماكن المزدحمة كالمواصلات العامة او فى محيط الأسرة أو العمل** |  | |
| **19- عدوى من الجروح** |  | |
| **20- عن طريق أكل ملئ بالدهون** |  | |
| **21-عن طريق الحجامة** |  | |
| **22-** **إستعمال الحمامات ودورات المياه العامة** |  | |
| **23-** **استخدام أجهزة الهاتف العامة** |  | |
| **24-**  **التجاور فى قاعات الدراسة** |  | |
| **25-عن طريق ملامسة الذباب** |  | |
| **[10]هقولك بعض الأعراض وتقولي رأيك فيها**  **1=موافق 2= غير موافق 3= محايد (قد يحدث وقد لا يحدث) 4 = لا أعرف** |  | |
| **الأعراض** |  | |
| **1-من أعراض المرض ارتفاع في درجة الحرارة** |  | |
| **2-من أعراض المرض اصفرار في العين والجلد** |  | |
| **3-من أعراض المرض الام في الجهة اليمنى من البطن** |  | |
| **4-من أعراض المرض لون البول داكن** |  | |
| **5-من أعراض المرض ألام بالمفاصل** |  | |
| **6-من أعراض المرض التعب من أقل مجهود** |  | |
| **7-لا يوجد أعراض للمرض** |  | |
| **[11]هقولك بعض المضاعفات وتقولي رأيك فيها؟**  **1=موافق 2= غير موافق 3= محايد 4= لا أعرف** |  | |
| **المضاعفات** |  | |
| **1-تليف في الكبد** |  | |
| **2-سرطان الكبد** |  | |
| **3-دوالي المرئ** |  | |
| **4-استسقاء بالبطن (ميه)** |  | |
| **5-التهاب مزمن في الكبد** |  | |
| **6-فشل كبدى ( الخمول، الكسل، همدان، ضعف، هبوط، ارهاق، تعب،** |  | |
| **7- مشاكل نفسية ( اكتئاب، عصبية، قلق)** |  | |
| **[12]هقولك بعض العبارات وتقولي رأيك فيها؟1=موافق 2= غير موافق 3= محايد (قد يحدث وقد لا يحدث)4= لا أعرف** |  | |
| **1-يستحسن تطعيم مرضى الكبد الوبائي سي بتطعيم الكبد الوبائي أ وتطعيم الكبد الوبائي ب** |  | |
| **2- مرضى الكبد الوبائي سي يمكن أن يعيشوا سنين عديدة دون أن يعرفوا أنهم مرضى بالفيروس** |  | |
| **3-استخدام إبر أو أي آلات حادة جديدة لم يسبق استخدامها ممكن أن يقلل من التعرض لمرض الكبد الوبائي سي** |  | |
| **4-العلاج الصحيح والناجح يؤدي إلى اختفاء فيرس الكبد الوبائي سي من دم المريض تماما** |  | |
| **5-بعض العلاج من الفيروس سي مثل الإنترفيرون قد يؤدي إلى بعض المضاعفات مثل الإكتئاب** |  | |
| **استمارة الرغبة للتغيير والتغيير السلوكي** |  | |
| **[13] هل يمكن ان تقوم او انك تقوم فعلا بتلك الممارسات؟ 1= بالطبع لا 2= ممكن لا 3= ممكن نعم 4= بالطبع نعم** |  | |
| **العبارات** |  | |
| **1- تطلب من الحلاق تغيير موس الحلاقة امامك** |  | |
| **2- تطلب من الحلاق الحلاقة بماكينة الحلاقة الخاصة بك** |  | |
| **3- التأكد من طبيب الأسنان من ان الألات معقمة لعلاج الأسنان** |  | |
| **4-ابلاغ طبيب الأسنان بأن عندك الكبد الوبائي سي (فى حالة اصابتة بالفيروس س)** |  | |
| **5- استخدام فرشاة الأسنان دون المشاركة مع أحد من أفراد الأسرة** |  | |
| **6- استخدام المقص اوالقصافة عند قص الأظافر دون المشاركة مع أحد من أفراد الأسرة** |  | |
| **7- استخدام لوفة خاصة دون المشاركة مع اى فرد من الأسرة اثناء الأستحمام** |  | |
| **8- استخدام دبابيس الطرحة دون المشاركة مع أفراد الأسرة (للسيدات المحجبات)** |  | |
| **[14] يا ترى لو حد من أفراد اسرتك عرض عليك الآتي ممكن تستخدمه؟1= بالطبع لا 2= ممكن لا 3= ممكن نعم 4= بالطبع نعم** |  | |
| **1-ملابسه** |  | |
| **2-أدواته للحلاقة** |  | |
| **3-فرشة أسنانه** |  | |
| **4-أدواته للشرب أو الطعام دون غسيل** |  | |
